# AdaBoost based Random forest model for Emotion classification of Facial images

**DOI:** 10.1016/j.mex.2023.102422

**Published:** 2023-10-14

**Authors:** Kumari Gubbala, M. Naveen Kumar, A. Mary Sowjanya

**Affiliations:** a Department of CSE, CMR Engineering College, Hyderabad, Telangana, India; b Department of CS&SE, Andhra University College of Engineering (A), Visakhapatnam, Andhra Pradesh 530003, India

**Keywords:** AdaBoost and RandomForest Classifier, Sentiment analysis, Emotion classification, Facial emotion, 2-dimensional discrete ortho-normal stock well transformation (DOST), AdaBoost, Random forest

## Abstract

Posting of visual data in the social network has now become a common trend. Mainly, users are posting selfies or facial images over the social media that depict various moods at different instances. This has attracted the attention of researchers to come up with facial expression mining from social media images. Aim of the present work is to improve the performance of emotion analysis in a more efficient way in terms of accuracy and reliability. Developing new strategies for carrying out emotion analysis on posts containing images in social media. In this work, a novel model has been presented that focuses on transformed features for the purpose. Six distinct sentimental emotion classes (labeled 0 through 5) are considered in this work. They are 0: Sad, 1: Fear, 2: Awful, 3: Happy, 4: Surprised, 5: Satisfied. This model consists of three major stages: Feature extraction, Feature selection, and Class labeling.

• This work incorporates the use of 2D Ortho-normal Stockwell Transformation (DOST) method is used for feature extraction of facial images.
• Following the feature extraction model, feature selection is implemented through ‘bi-variate t-test’.
• Finally, these selected features are subjected to a AdaBoost based Random Forest classifier for Emotion Classification(ARFEC) for the purpose of class labeling towards different classes of expression.

The Flickr8k, CK+ and FER2013 image databases are utilized for validating the efficiency of the developed ARFEC model. Analysis of results shows the effectiveness of ARFEC model with overall rates of accuracy of 89.5 %, 92.5 % and 89.5 % respectively for the databases taken. Performance of ARFEC model when compared with other existing methods such as Support Vector Machine and K-Nearest Neighbors yielded better results in terms of overall rate of accuracy.

## Graphical abstract

**Figure:**
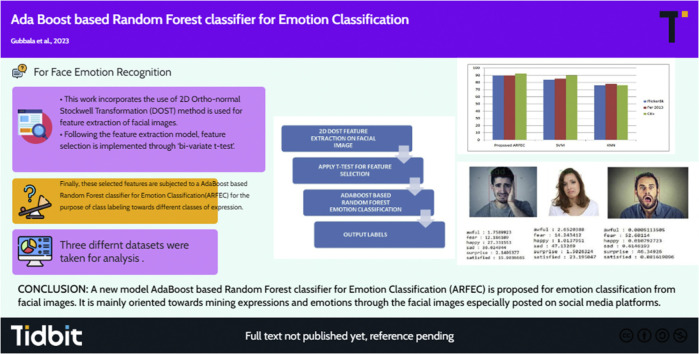

Specifications table

**Table:** 

Subject area:	Computer Science
More specific subject area:	Ensemble Machine Learning model
Name of your method:	AdaBoost and RandomForest Classifier
Name and reference of original method:	N.A
Resource availability:	N.A

**Method details**

## Introduction

It is well known that numerous meanings can be expressed by just a facial gesture. A human face is capable of conveying multiple emotions through different expression gestures. In the present age of social media, the same phenomenon is occurring through the digital exchange of facial expression images. The process of detecting emotion from facial gestures is known as Facial Emotion Recognition. The ability to detect emotions such as guilt, fear, and uncertainty can be useful to governmental organizations in the public sphere. Businesses also explore emotion recognition in order to enhance their financial performance [1].

A viewer is always curious to analyze the mood of the other person through his/her facial image posts. An automated system that can infer expression and emotions out of human faces posted on social media platforms serves the purpose. Researchers have picked up this challenge and presently showing active participation in this type of research work. This can have important applications in various fields like medical, depression management, teaching and learning, business tactics, etc.,.

## Literature review

Sentiment mining is performed on normal data on the social media and it is validated at the VLSP 2016 evaluation campaign [2]. The mining task was confined to subject specific domains. For this, low cost ensemble classification model was proposed making use of random forest [3], support vector machine [4], and the naive Baye's [5] techniques. Approximately 70 % accuracy has been reported for this work. On the other hand a Convolutional Neural Network (CNN) is used as a tool for performing facial expression mining [6]. Initially, Regions Of Interest (ROIs) are extracted from input image. Two eye points are kept as the reference centers. Local mouth region, eye region and filtered ROI's are fed to the CNN for further feature extraction and classification task. Six different types of facial expressions are considered for validation of this model. The Extended Cohn-Kanade (CK+) database alone has been used for sample collection. Person specific expression mining from face image is proposed in [7]. This work proved to be helpful for HCI (human computer interaction) tasks. Although facial geometric alignment being one of the key issue n this work. They have used prototype based expression recognition. For this, SIFT features are extracted and similarity score is used for classification. Overall 87 % of accuracy is reported for this work on the JAFFE database and an rate of accuracy of 83 % has been reported on the GEMEP-FERA database. Linear caricatures features are considered for performing automatic expression recognition from human faces [8]. It is reported to be helpful in real time analysis of human emotions. Geometric and structure based features are generated and used along with line based mapping strategies for execution of the work. A specific measurement (disparity based) is also used to bring in robustness to the work. SVM has been efficiently used in three stages for the expression mining from social media face images [9].The first stage takes binarized combination inputs from seven distinct classes. The second stage involves twenty-one SVM models. Image processing has been used intensively for suitable feature extraction. The primary focus area made here is the muscular relative movements that brings in changes in the gradients.

The work is validated on three different databases through a k-fold cross validation mechanism. Support vector machine is also been used efficiently in [10]. The proposed FERS technique utilizes Haar-like traits from images using SQI filters. This enhances the rate of face detection first. SQI filters are well known for working efficient for varying lighting conditions. They have chosen the discrete cosine transform (2D-DCT) along with Gabor filtering for feature expression. Finally SVM is used as the classifier that categorizes the facial expressions into distinct classes.

Using external attributes like size, appearance, shape, and color, Habib et al. [11] analyze and classify dates manually. In addition to the time-consuming process, they note that there are quality risks and additional costs associated with this time-consuming post-harvest procedure. A key finding of the paper is that computer technology is underutilized in the cultivation of fruit and dates. A novel image processing algorithm that utilizes machine learning to automate date classification is presented by the authors through the extraction of color intensity and homogeneity features from date images. This paper demonstrates the algorithms' ability to assess individual and group date quality. This study reports an impressive 95 % accuracy rate, suggesting the potential for computer vision algorithms to improve date classification and sorting, relieving manual intervention.

Bagherzadeh andToosizadeh [12] Eye tracking systems are important components of Human Machine Interface (HMI) equipment, and this paper presents an algorithm for increasing eye tracking efficiency using multi-model Kalman filters.Multi-model Kalman filters employ multiple models for object estimation, aiming to boost efficiency and reduce estimation errors. Based on the typical behavior of the human eye, the algorithm first recognizes the initial eye position using the Support Vector Machine (SVM), and then uses a multi-model Kalman filter to predict the eye's position in the next frame using constant speed and acceleration models.

Tawsif et al. [13] This paper explores how physiological signals from biosensors can be leveraged to identify emotions of users. There is a differentiation between contact-based and contactless sensors, as well as an explanation of the ERS process. Results of various techniques are provided. Researchers interested in integrating emotion recognition into their systems can benefit from this review of 147 relevant articles from 2009 to 2021.

## Proposed method

The proposed model using different datasets which are given below:

1. Flickr-Faces-HQ Dataset (FFHQ)

Flickr-Faces-HQ (FFHQ) is a high-quality image dataset of human faces. The dataset consists of 70,000 high-quality PNG images at 1024 × 1024 resolution and contains considerable variation in terms of age, ethnicity and image background. The images were crawled from Flickr, thus inheriting all the biases of that website, and automatically aligned and cropped.

2. FER-2013

The data consists of 48 × 48 pixel grayscale images of faces. The faces have been automatically registered so that the face is more or less centered and occupies about the same amount of space in each image. The Facial Expression Recognition 2013 (FER2013) database was first introduced in the ICML 2013 Challenges in Representation Learning. This dataset contains 35,887 images of 48 × 48 resolution, most of which are taken in wild settings. This database was created using the Google image search API, and faces were automatically registered. The faces are labeled as any of the six cardinal expressions.

3. The Extended Cohn-Kanade Dataset (CK+)

The Cohn-Kanade (CK) database was released for the purpose of promoting research into automatically detecting individual facial expressions. Since then, the CK database has become one of the most widely used test-beds for algorithm development and evaluation. The extended Cohn–Kanade (known as CK+) facial expression database is a public dataset for action unit and emotion recognition. It includes both posed and non-posed (spontaneous) expressions. The CK+ comprises a total of 593 sequences across 123 subjects. The last frame of these sequences is taken and used for image-based facial expression recognition.

**Fig. 1:**
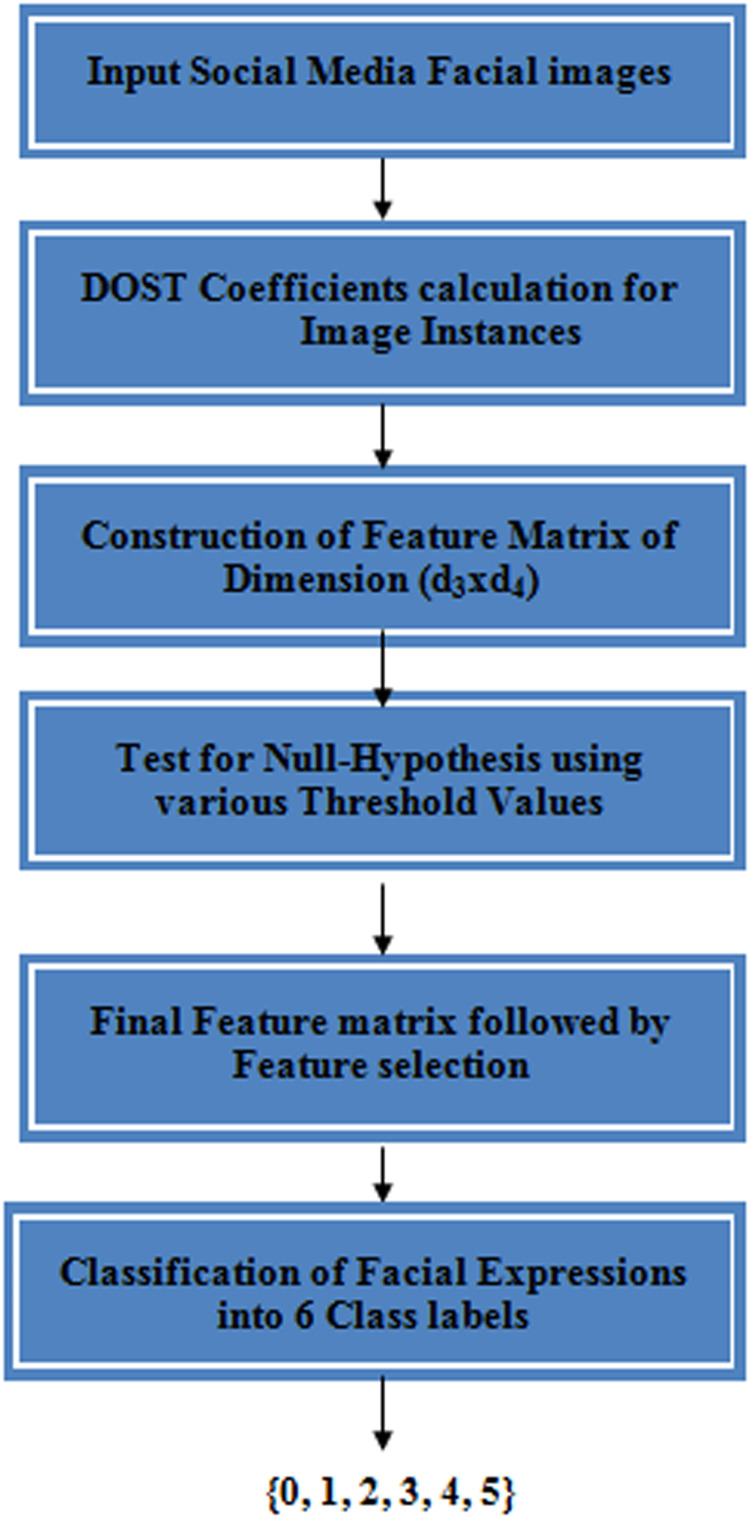
General overview of the proposed work.

2-Dimensional Discrete Ortho-normal Stock well Transformation (2D-DOST) is utilized for feature extraction from the social media facial gesture images in this work. AdaBoost based Random Forest classifier for Emotion Classification (ARFEC) model has been proposed. This model uses null hypothesis with bi-variate t-test for choosing prominent features out of the entire feature set. An AdaBoost based Random Forest classifier for Emotion Classification (ARFEC) model is utilized for the purpose of classifying the facial expression into respective categories. The general overview of the proposed ARFEC model is shown in Fig. 1.

**Fig. 2:**
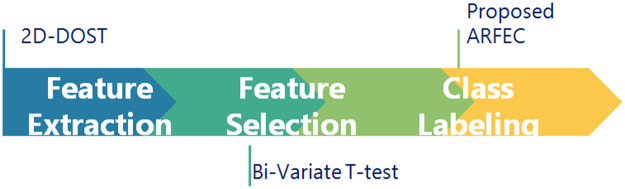
Stages in the proposed ARFEC model.

This model consists of three major stages: Feature extraction, Feature selection, and Class labeling shown in Fig. 2.

### Feature extraction

This phase utilizes the mechanism of two-Dimensional Discrete Ortho normal Stockwell transformation (DOST). It is a multi-scale strategy that focuses on extracting texture related features at the pixel level of an image as per the S-transformation and works close to the wavelet transformation [14]. Linear mode of scaling for the frequency data is conserved in this technique, thus making it suitable for facial expression classification. Although limitation of the same is the higher value for computational cost that arises due to the redundancy property. To overcome this, certain preliminary functions are utilized by DOST model. Thus, this improved DOST model has better computational cost and storage efficiency compared to the earlier model.

### Feature selection and class labeling

There are six distinct sentimental expressions classes (labeled 0 through 5) considered in this work. Those are:

• 0: Sad,
• 1: Fear,
• 2: Awful,
• 3: Happy,
• 4: Surprised,
• 5: Satisfied.

Null hypothesis validation is conducted on six distinct set of samples (as there are six distinct classes under consideration, say 0; 2... 5). The validation result indicates the correctness value for the hypothesis. This in turn, triggers the samples among the class distributions and could become able to indicate whether there exist differential evidences among them or not. We should not that an incorrect decision for the hypothesis may result in insufficient difference among the samples distributions in different classes. The trick used for conducting the test relies on the strategy that it is to be cascaded. It means that among the six classes of samples initially, it is performed on 0 versus 1; 2; 3; 4; 5;. Then it is followed by the test on 1 versus 0; 2; 3; 4; 5; and so on.

The AdaBoost based Random Forest classifier for Emotion Classification(ARFEC) model is used for the classification and labeling. The random forest ensemble classifier model provides satisfactory efficiency in terms of rate of accuracy. Bootstrapping is followed to create distinct classifiers in this ensemble through bagging. Intermittent feature points are chosen and the decisions are done at nodes where split strategy is needed. Rate of error for this ensemble relies directly on the chosen feature points and their quantity. Multiple numbers of tree predictors are engaged where they rely on the randomized sample vectors with respect to single distribution in the single forest. With a training sample set and the randomized vector individual trees are made. After successful creation of the trees, the classification hypothesis is placed based on voting expert strategy made on each of the class.

AdaBoosting is a popular strategy. Which provides flexibility for addition of multiple weak classification methods with higher rates of error. It generates an integrated hypothesis for which the rate of error becomes reduced [15], Assuming a binary classification scenario, taking training samples D = (D1i, D2i). D1i and D2i are representing the vector values and the class labels (0,1) respectively. Every training sample vector is associated with a weight value pretending the self probability. The weak classifier gets training iteratively with the weighted training sample vectors. Every successive iteration of the training reduces the corresponding error rate for that weak classifier with updated weights. Finally, linear combination of the hypothesis calculated for each round provides the concluding decision.

## Experimental evaluation

**Fig. 3:**
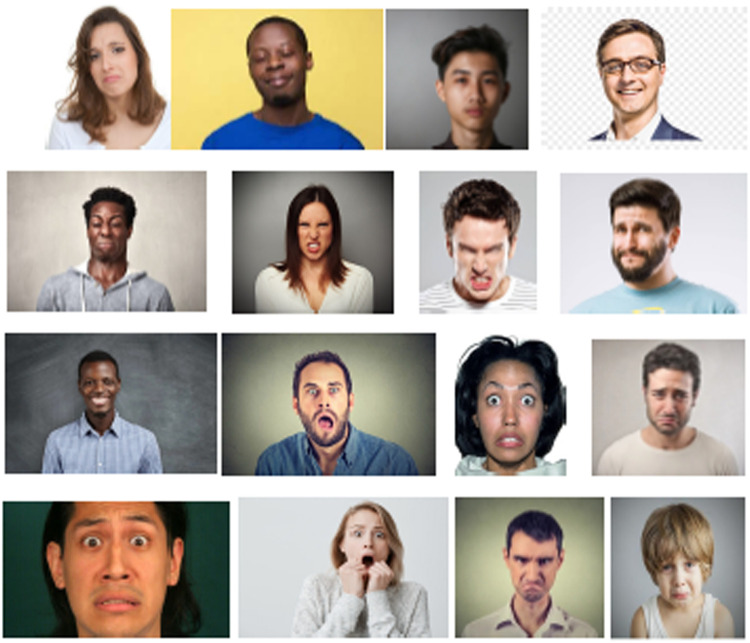
Various facial expression samples.

The proposed ARFEC model is validated on 400 distinct facial images with reference to social media posts only. These images are drawn on equal sampling basis among the six distinct classes of expressions. The samples for various classes of facial expressions are shown in Fig. 3.

**Fig. 4:**
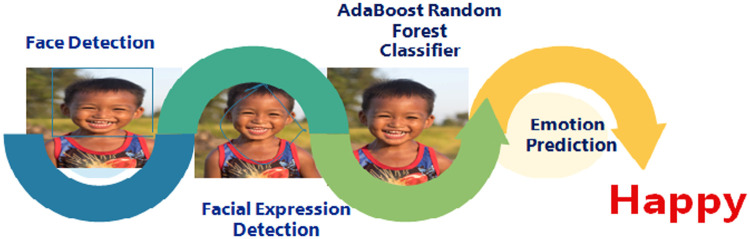
Steps for emotion prediction.

**Fig. 5:**
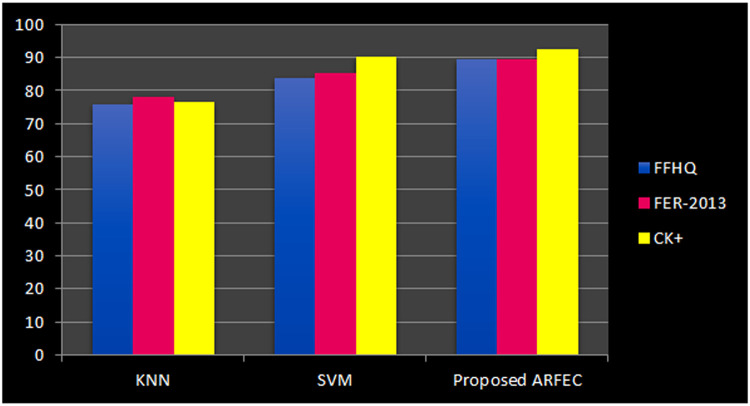
Accuracy metrics for our proposed ARFEC model.

The dimensions for the input samples are standardized to be 128 × 128. This results in generation of same number of (128 * 128) coefficients for respective initial feature matrix. Suitable feature choices are made with the help of reduced feature matrix. Various values for threshold are considered and respective set of feature are generated. Thus different sub-datasets are obtained. They are now fed to the classification labeling stage. The steps in emotion prediction and the accuracy metrics for the proposed ARFEC model are given below in Fig. 4, Fig. 5.

The proposed ARFEC model is compared with two other competent models for the purpose of facial emotion mining from social media posts. The two competent models chosen for the purpose are SVM (support Vector Machine), an KNN (K Nearest Neighbor).

**Fig. 6:**
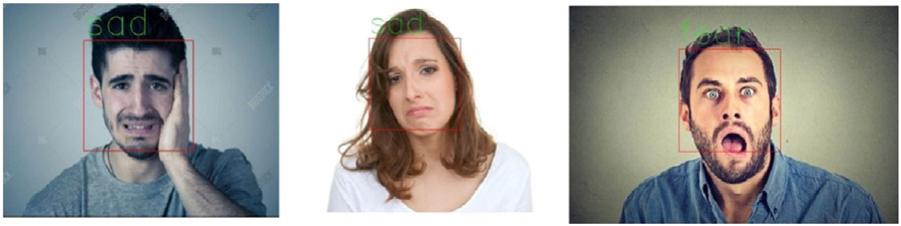
Sample outputs obtained from the proposed method.

Here, a k-fold cross validation mechanism (k = 10) is followed for each of the experiment (each sub-dataset obtained above).For the purpose of classification, random forest comprising of ten, twenty, thirty, forty, fifty, and sixty trees are considered separately. All the trees with depth three are taken. The key parameters are considered for evaluation of results based on F-measure and overall best measure is obtained when the number of trees considered are forty. Maximum rate of accuracy with a value of 89.5 %, 92.5 % and 89.5 are computed during this validation process for the three databases (FFHQ [16], CK+ [17] and FER2013 [18]) .From the above graph, it can be noticed that the proposed ARFEC model outperforms the other two models. KNN showed an accuracy of 75.8 % while SVM showed 83.75 %, while the proposed ARFEC model showed 89.5 for FFHQ database. For FER-2013 database, KNN showed an accuracy of 78.0 % while SVM showed 85.0 %, proposed ARFEC model showed 89.5. Similarly for CK+ database, KNN showed an accuracy of 76.3 % while SVM showed 90.0 %, proposed ARFEC model showed 92.5. This is observed pretty well for the databases referred in this work. In Fig. 6., the sample outputs obtained through the Implementation of the proposed method is shown. From the dataset, the sample outputs for facial expressions give sadness, sadness, and fear depending on its emotion score for each emotion [19].

## Conclusions

A new model AdaBoost based Random Forest classifier for Emotion Classification (ARFEC)is proposed for emotion classification from facial images. It is mainly oriented towards mining expressions and emotions through the facial images especially posted on social media platforms. The method utilizes efficient image transformation model DOST for pooling the face features. Refined choice among pool of features is well accomplished with the help of hypothesized technique. Robust classifier like the random forest approach along with AdaBoost is finally used for the purpose of classifying different facial expressions. The method considers six distinct classes of expressions. Experimental evaluations are performed on samples drawn from three benchmark databases. Comparison of the proposed model with two other competent methods is made and efficacy of the same is observed to be satisfactory. This can be further extended to explore other social media platforms such as Face book, Instagram, Reddit etc. for emotion analysis.

## Ethics statements

The authors declare that informed consent was obtained from participants or that participant data has been fully anonymized, and b) the platform(s)’ data redistribution policies were complied .The images were used in this study are available in databases of social media posts collected for analysis.

## Declaration of Competing Interest

The authors declare that they have no known competing financial interests or personal relationships that could have appeared to influence the work reported in this paper.

## Data Availability

The data that has been used is confidential.
